# Supplementary figures and images for: Mapping the temporal transcriptomic signature of a viral pathogen through CAGE and nanopore sequencing
Source: PLoS One. 2025 Apr 15;20(4):e0320439. doi: 10.1371/journal.pone.0320439 (PMC11999163; doi:10.1371/journal.pone.0320439)

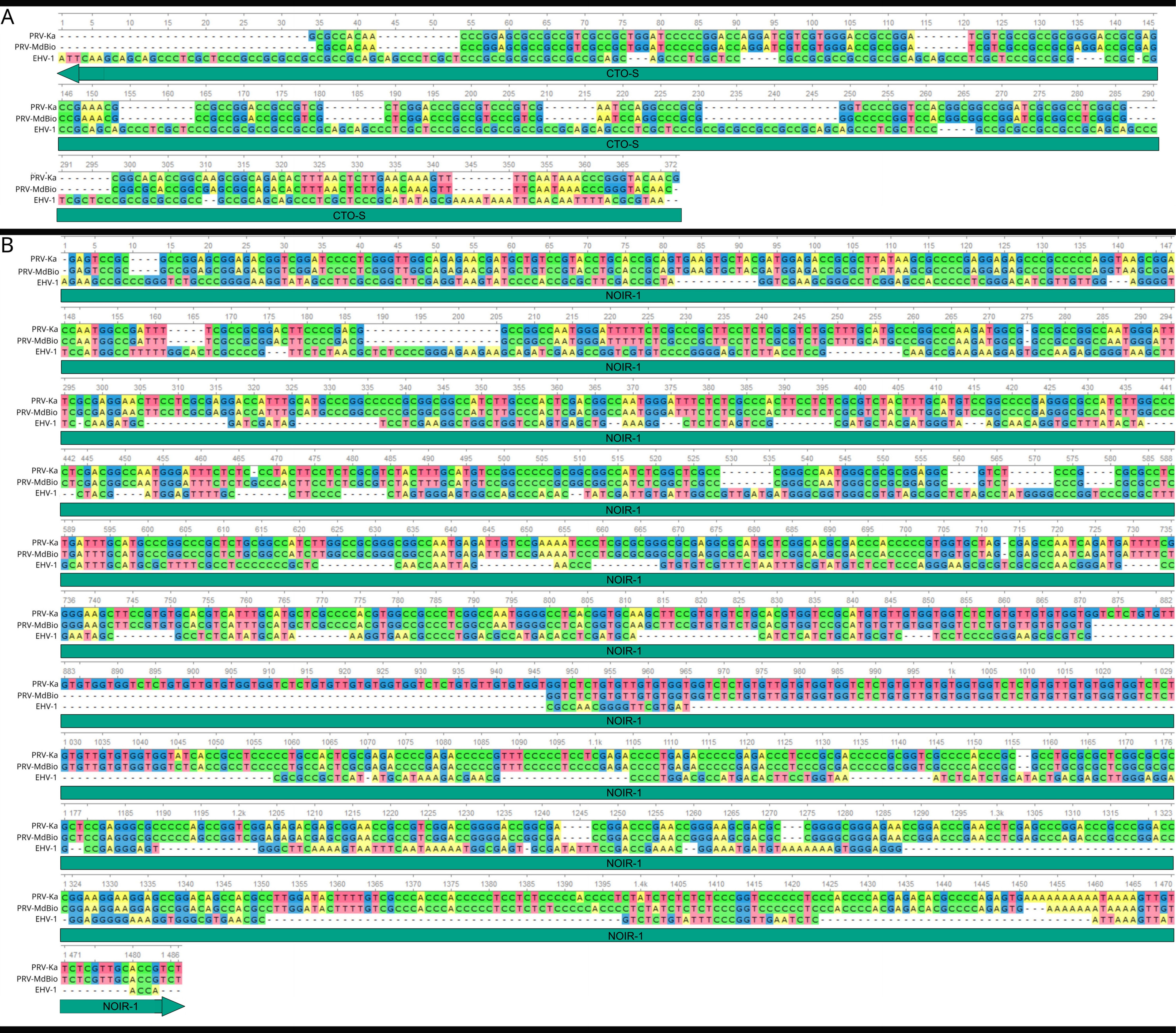

Supplement: S1 Figure — This illustration compares the sequences of raRNAs [(A) CTO-S; (B) NOIR] from EHV-1 with those of two PRV strains (Kaplan and MdBio). (JPG) [file pone.0320439.s001.jpg]

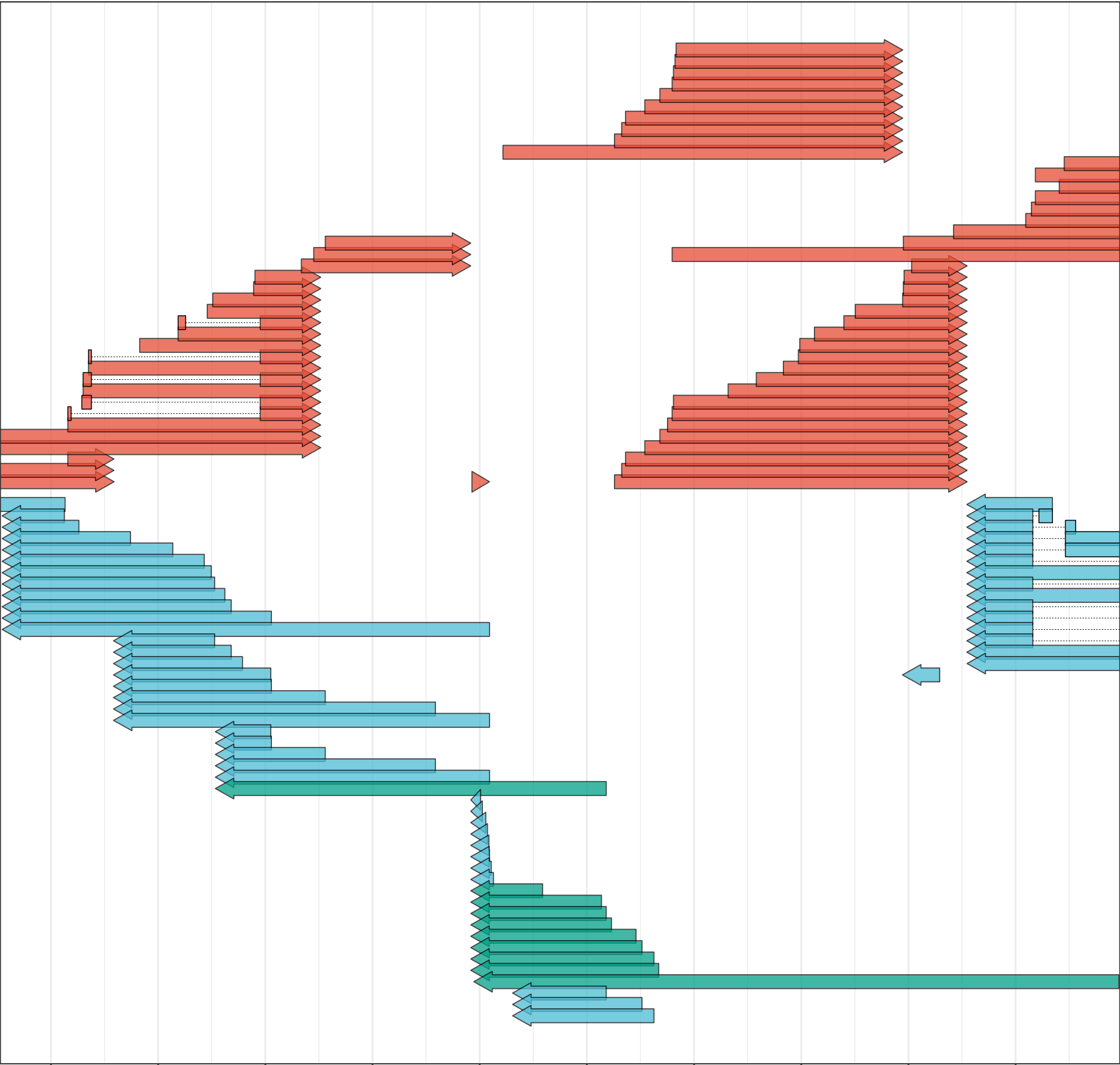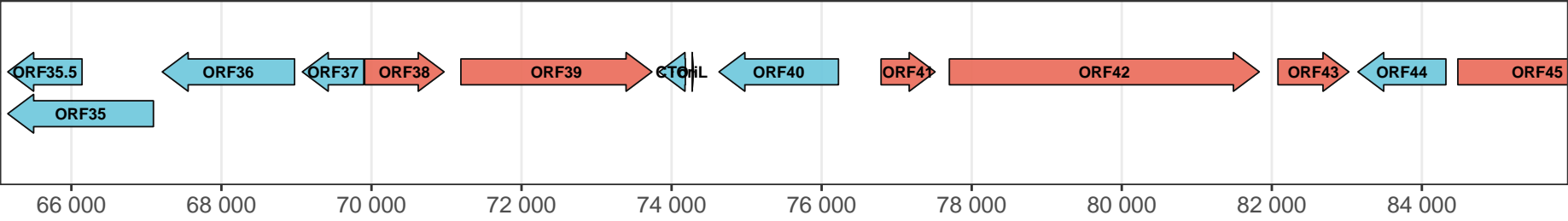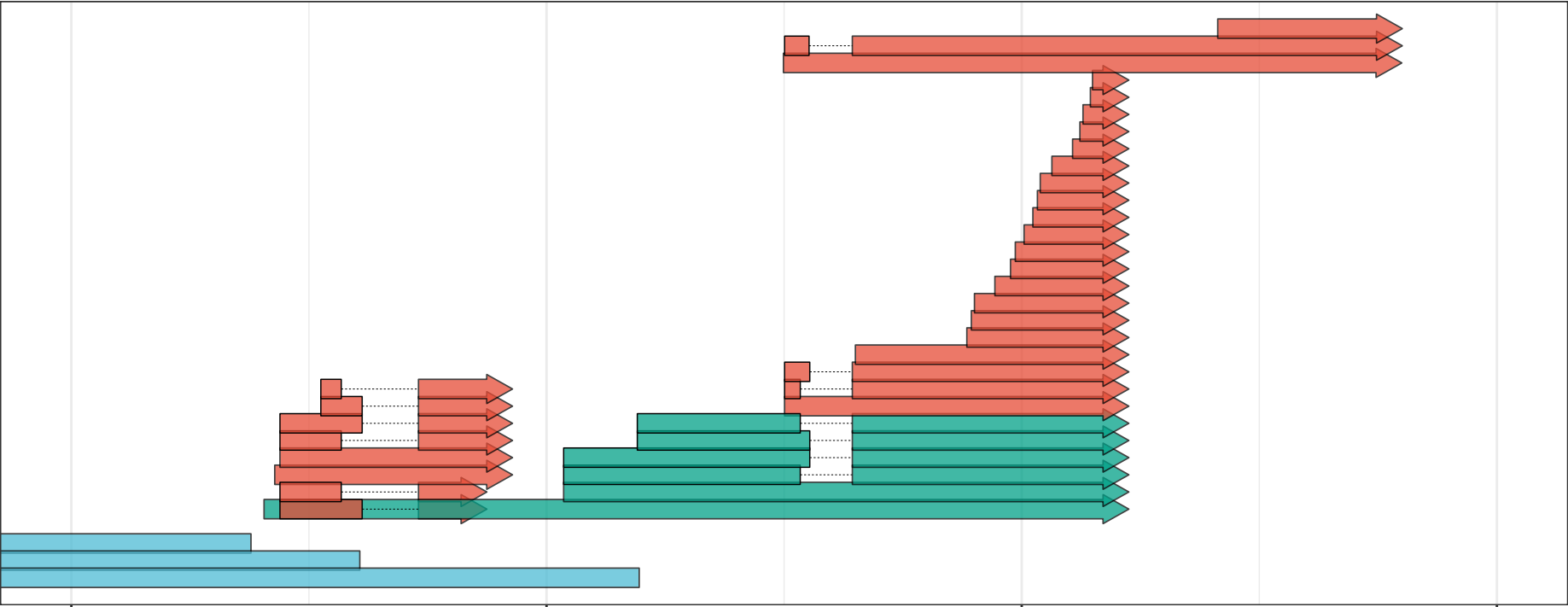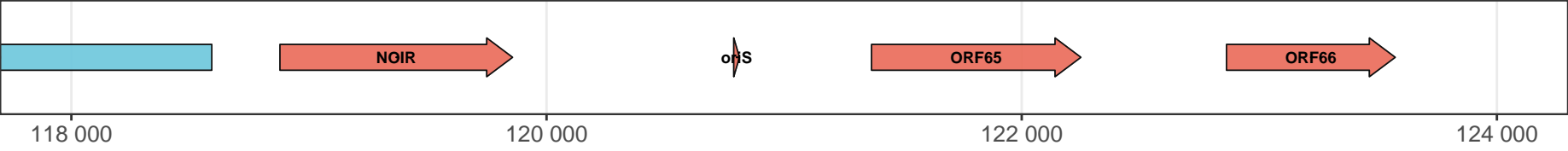

Supplement: S2 Figure — The OriL (upper panel) and OriS (lower panel) replication origins of equid alphaherpesvirus 1 and the genes located in their surrounding regions are shown. In the figure, transcripts and genes from the forward strand are shown in red, while those from the reverse strand are shown in blue. The replication-associated RNA (raRNA) molecules overlapping the replication origin are highlighted in green, while the Ori site is highlighted in purple. (PDF) [file pone.0320439.s002.pdf]

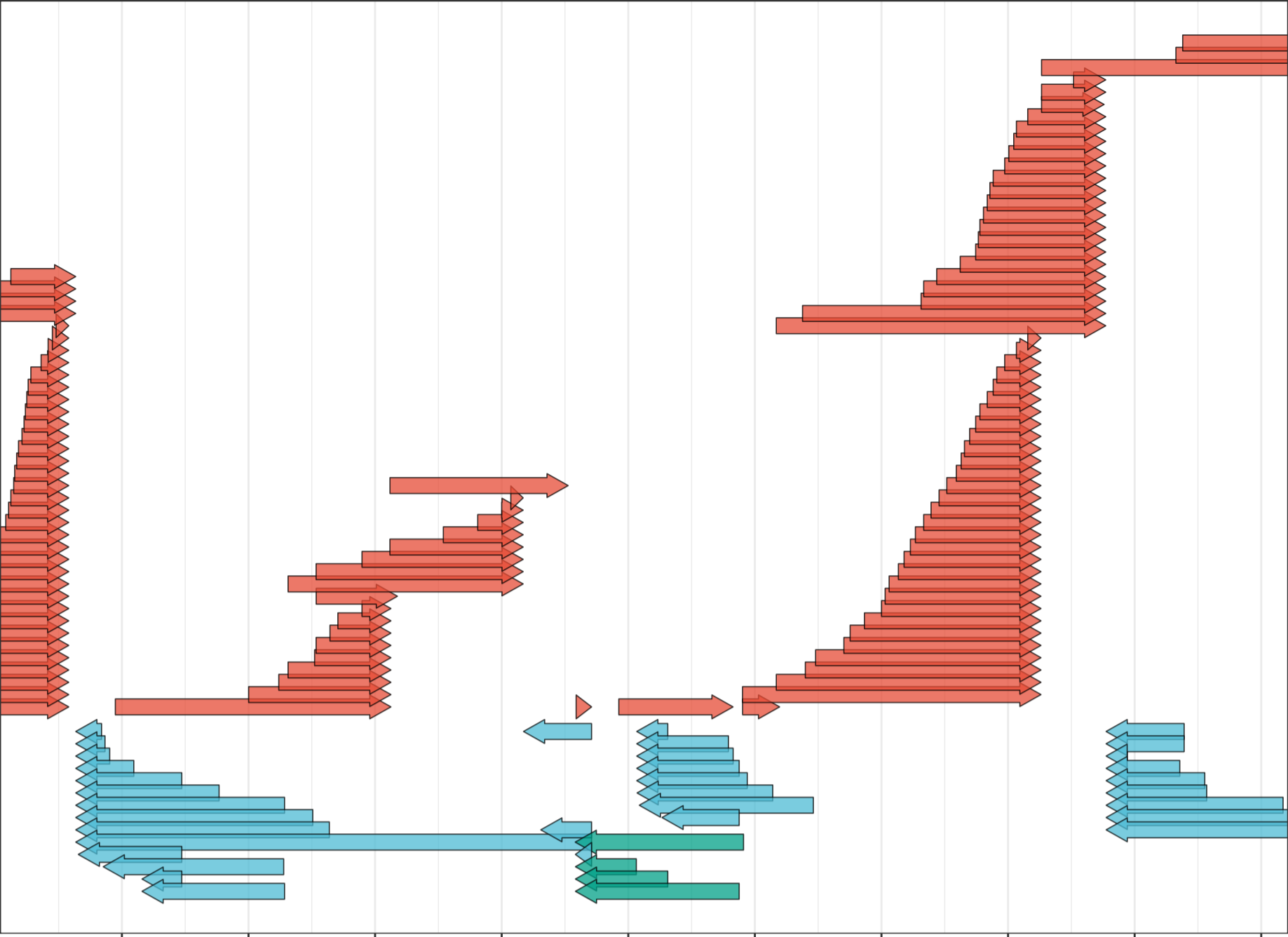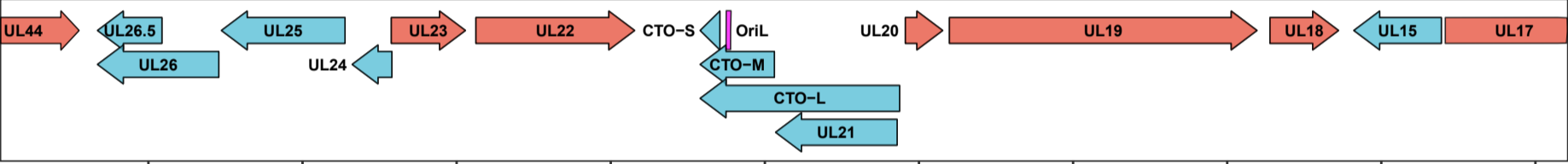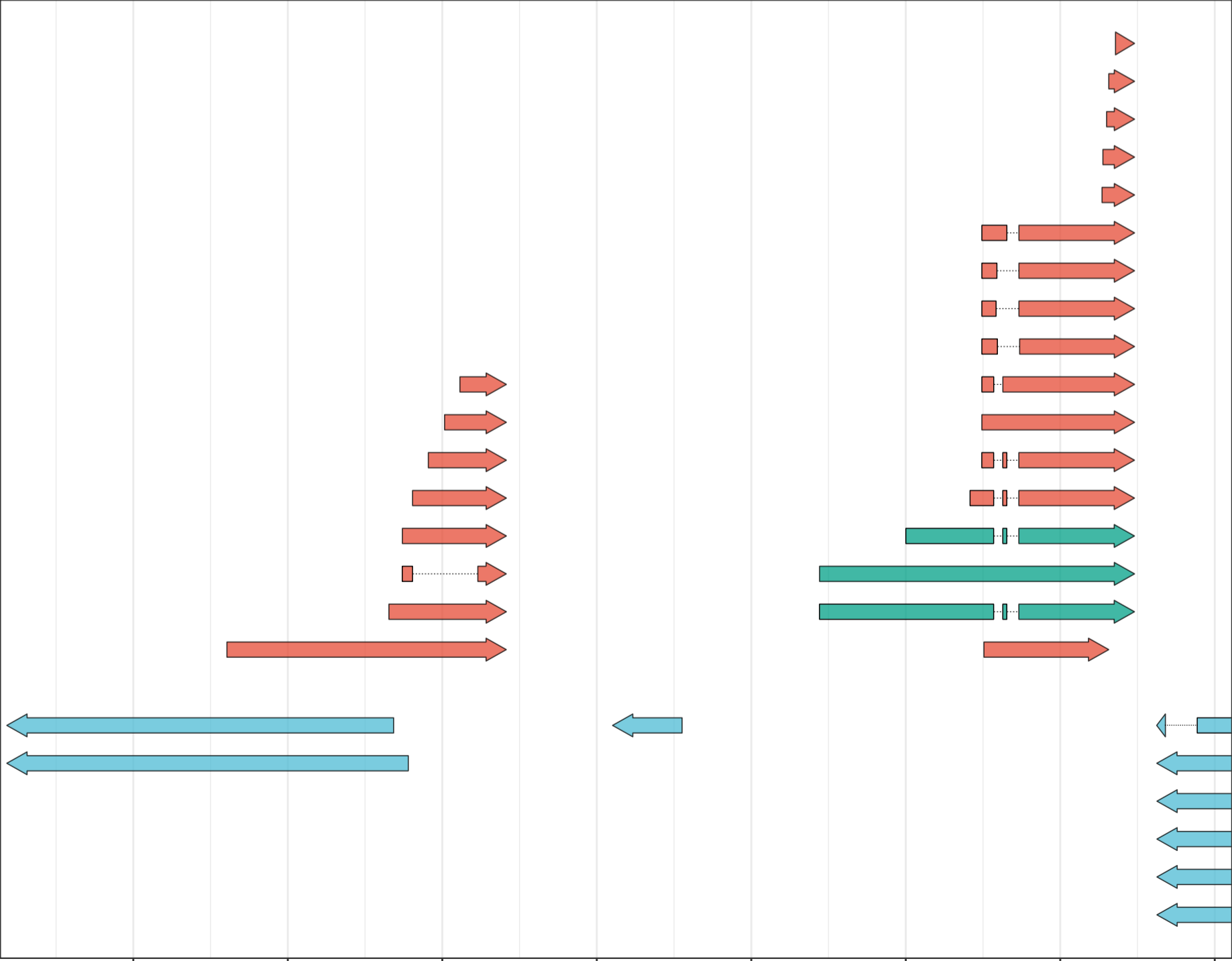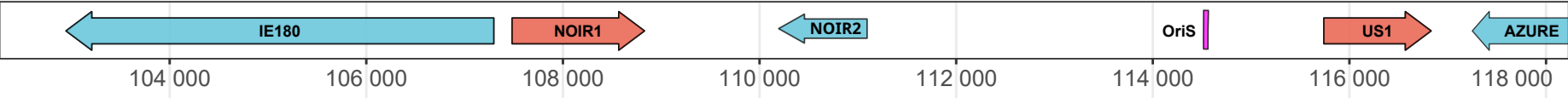

Supplement: S3 Figure — The OriL (upper panel) and OriS (lower panel) replication origins of pseudorabies virus, along with the genes in their surrounding regions, are depicted. In the figure, transcripts and genes on the forward strand are displayed in red, while those on the reverse strand appear in blue. The replication-associated RNA (raRNA) molecules that overlap the replication origin are highlighted in green, while the Ori site is marked in purple. (PDF) [file pone.0320439.s003.pdf]

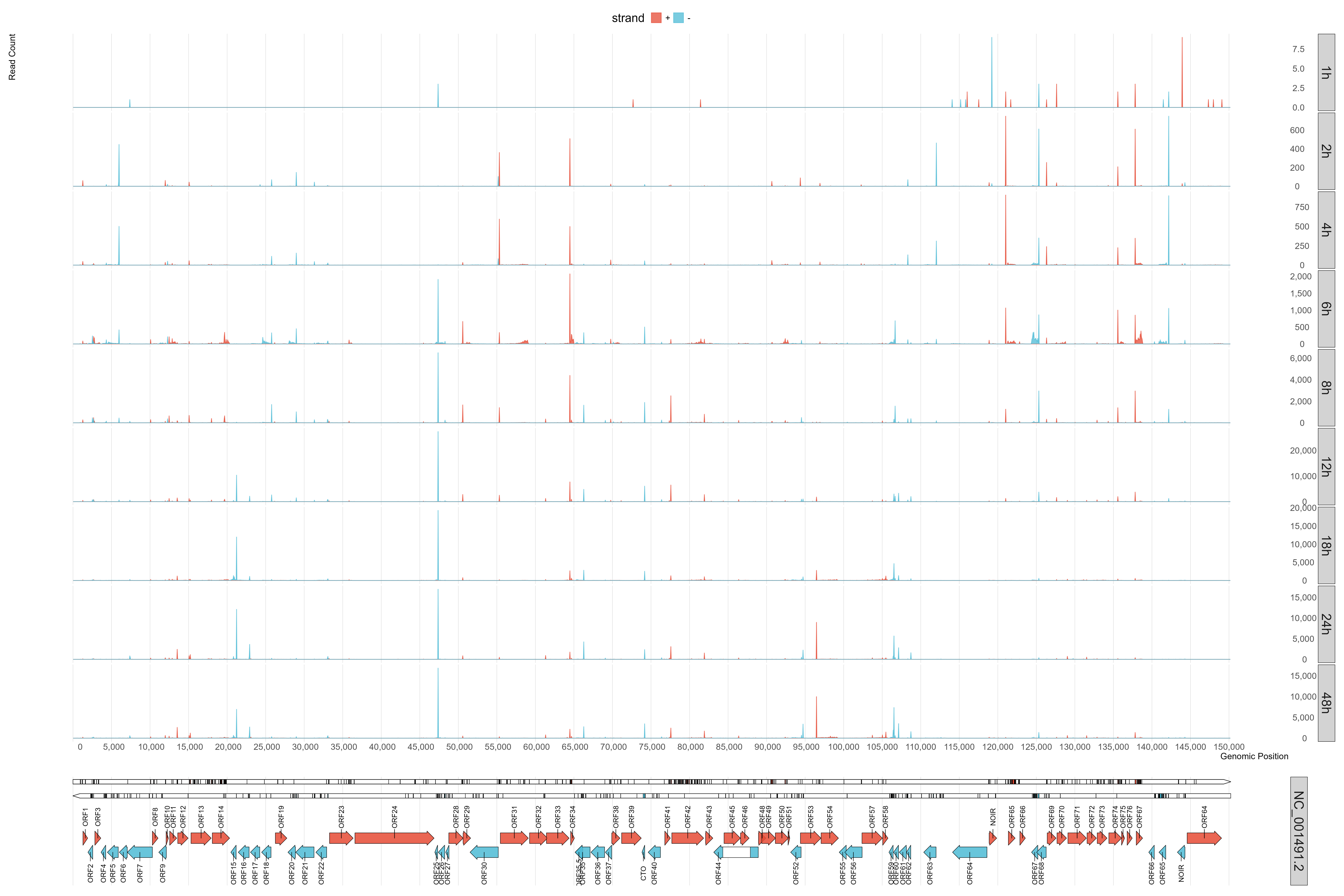

Supplement: S4 Figure — Similar to Figure 1, this plot shows the 5’-end distribution along the viral genome for each time-point group. The mean values for each time-point group were calculated and merged into 50-nt sized bins for visualization. Each facet corresponds to a time-point group, and the y-axis scale is determined independently (scale=“free_y” in ggplot). (JPG) [file pone.0320439.s004.jpg]

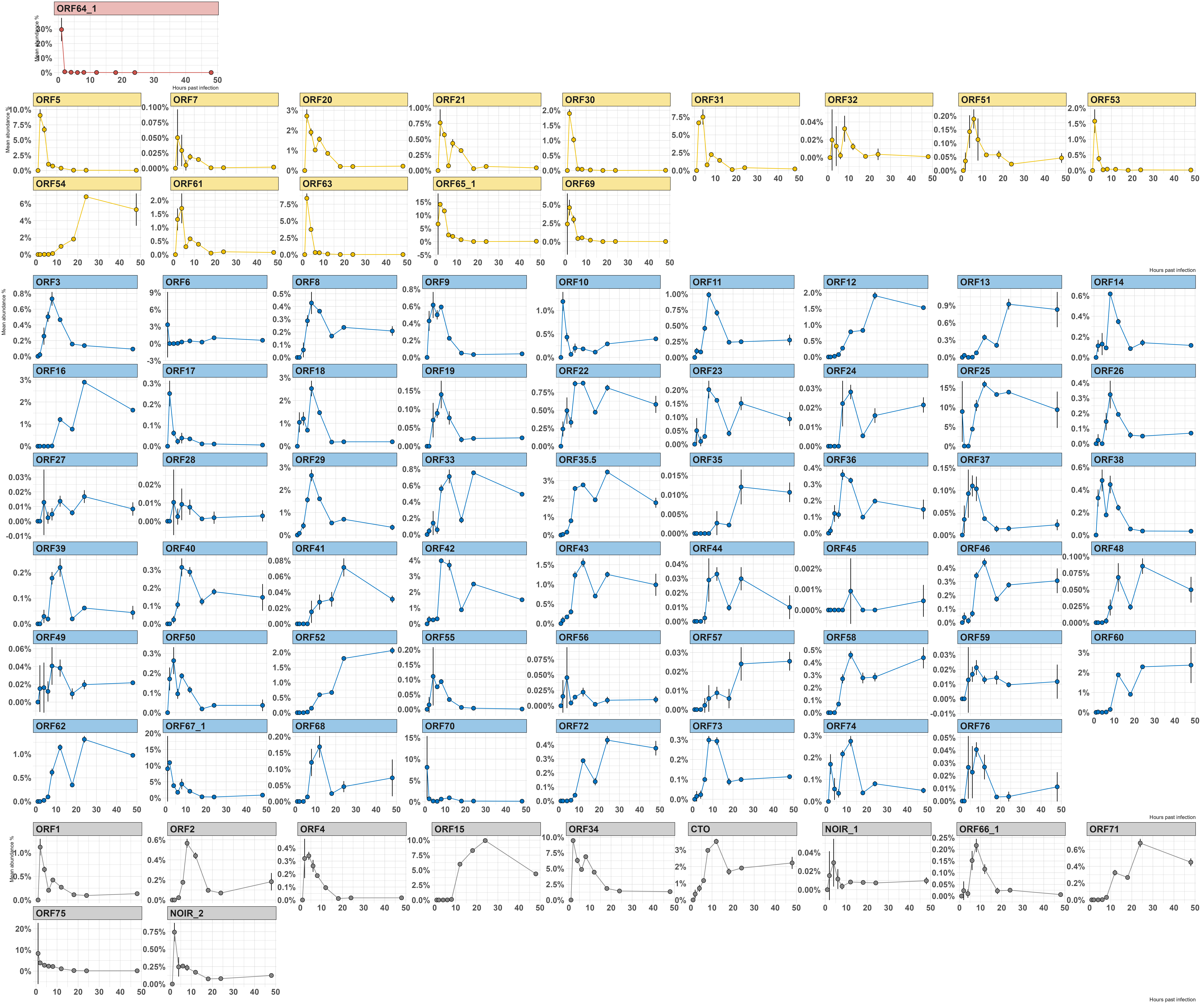

Supplement: S5 Figure — This figure illustrates the kinetic profiling of canonical EHV-1 TSSs, utilizing the total viral read counts per sample for normalization. The analysis included only those reads that aligned with the canonical TSS of genes at their 5’-ends (allowing a deviation of +/- 10 nucleotides). The mean values are represented as points, and standard deviations (SD) as lines, plotted on the y-axis as the ratio of TSS abundance for each gene. The x-axis represents time post-infection (hours). The panels are color-coded based on kinetic transcription phases: blue for immediate early (IE), orange for early (E), green for late (L), and red for unknown phases. This provides a visual distinction among different TSS dynamics throughout the infection. (JPG) [file pone.0320439.s005.jpg]

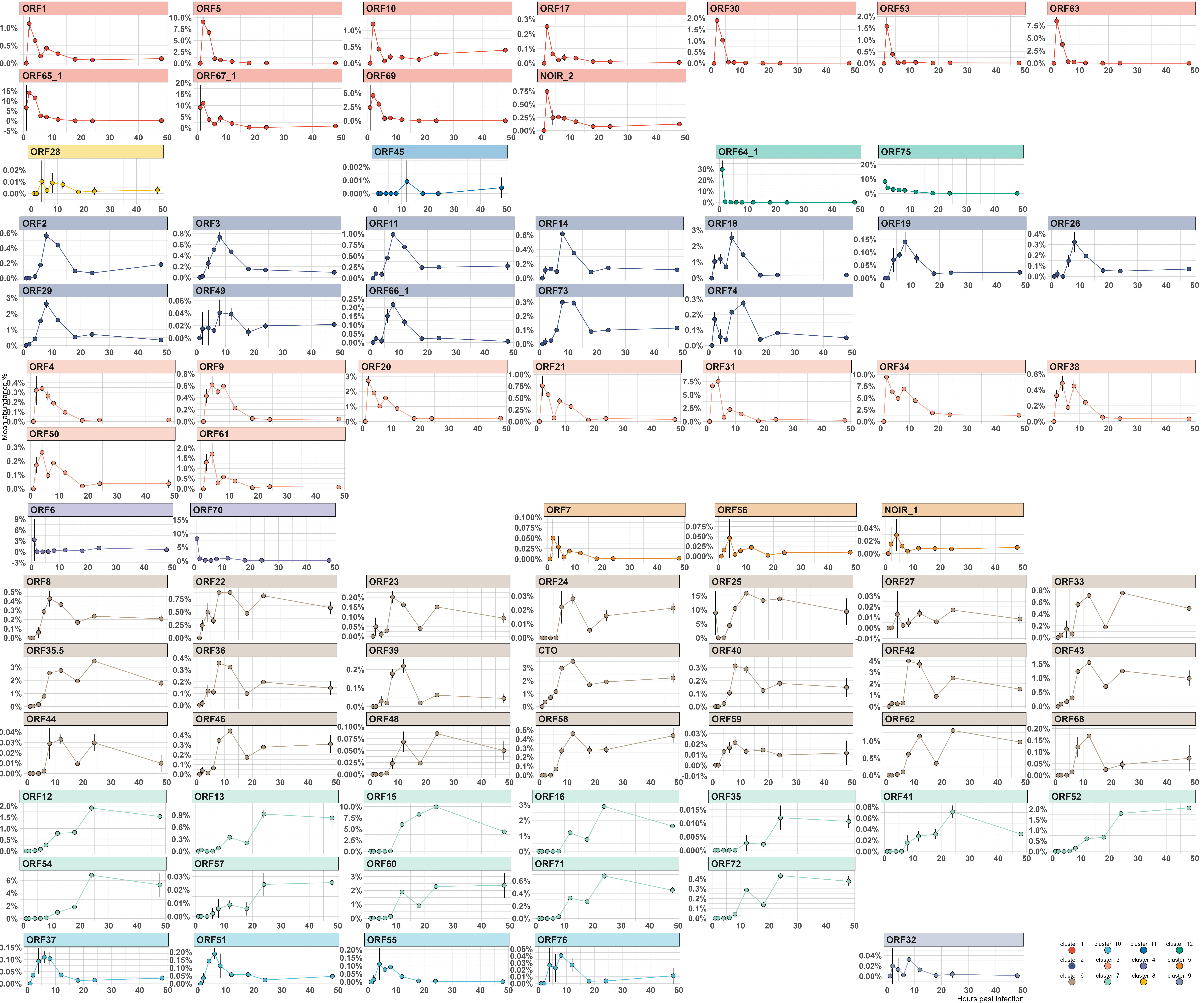

Supplement: S6 Figure — This figure illustrates the kinetic profiling of canonical EHV-1 TSSs, utilizing the total viral read counts per sample for normalization. The analysis included only those reads that aligned with the canonical TSS of genes at their 5’-ends (allowing a deviation of +/- 10 nucleotides). The mean values are represented as points, and standard deviations (SD) as lines, plotted on the y-axis as the ratio of TSS abundance for each gene. The x-axis represents time post-infection (hours). Each cluster is colored according to its de novo kinetic cluster membership. The color-coding for the clustering is shown in the bottom right panel. This figure provides a visual distinction among different transcriptional dynamics, according to the gene’s relative TSS abundance throughout the infection. (JPG) [file pone.0320439.s006.jpg]

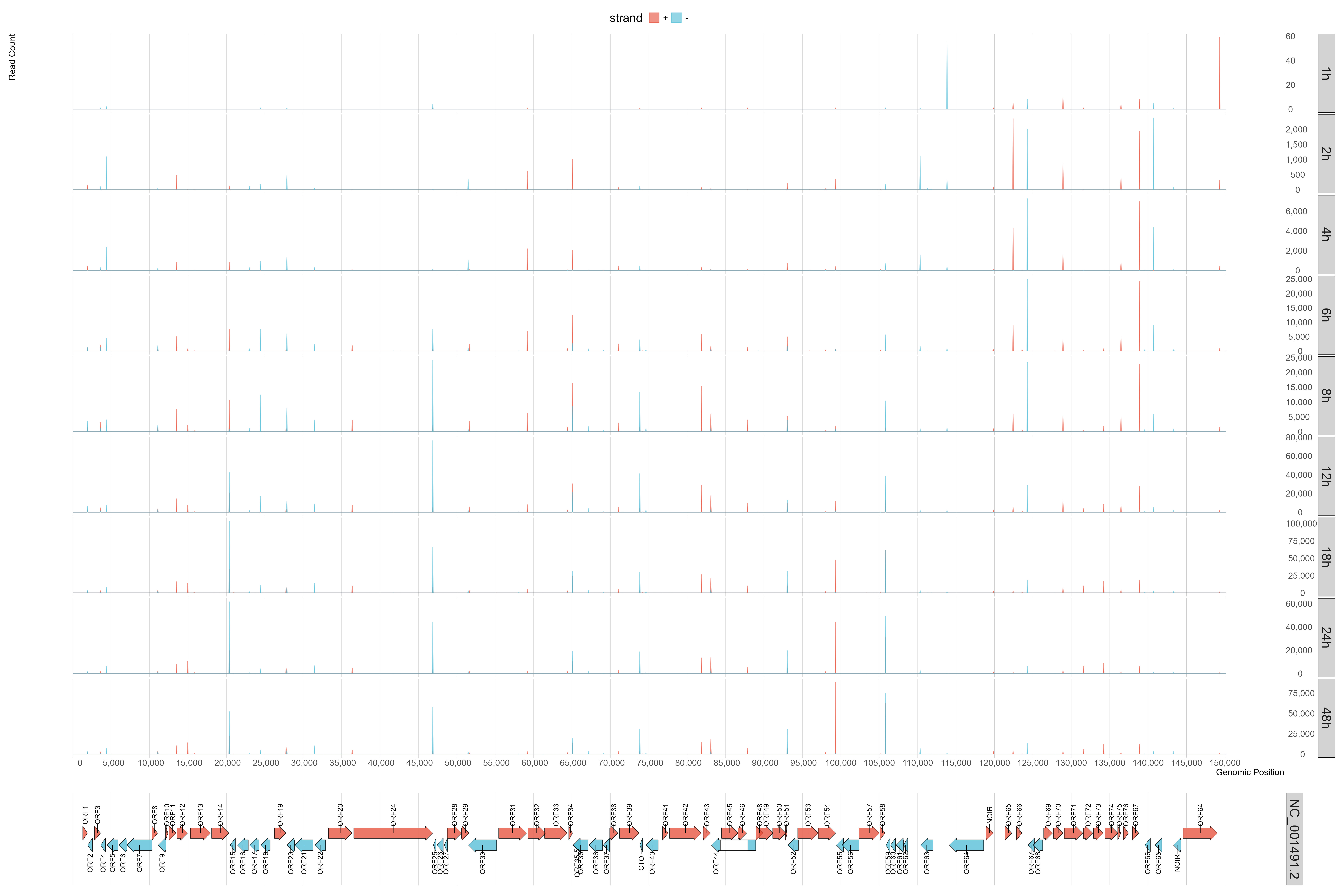

Supplement: S7 Figure — Similar to Figure 2, this plot shows the 5’-end distribution along the viral genome in each time-point groups. The mean values for each time-point group was calculated and merged into 50-nt sized bins for the visualization. Each facet represents a time-point group, with the y-axis scale set independently using scale=“free_y” in ggplot. (JPG) [file pone.0320439.s007.jpg]

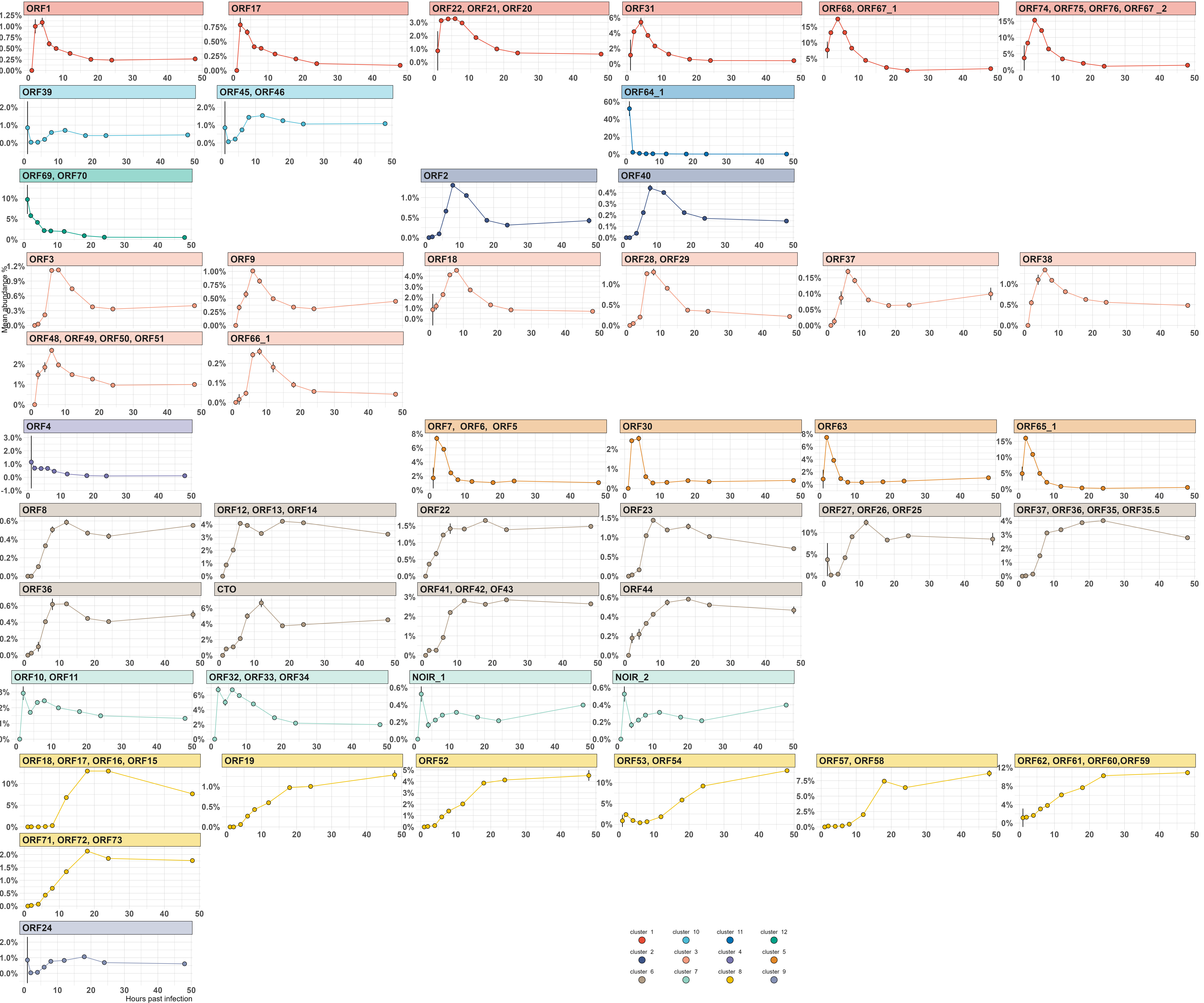

Supplement: S9 Figure — This figure illustrates the kinetic profiling of canonical EHV-1 TESs, utilizing the total viral read counts per sample for normalization. The analysis included only those reads that aligned with the canonical TSS of genes at their 5’-ends (allowing a deviation of +/- 10 nucleotides). The mean values are represented as points, and standard deviations (SD) as lines, plotted on the y-axis as the ratio of TES abundance for each gene. The x-axis represents time post-infection (hours). Each cluster is colored according to its de novo kinetic cluster membership. The color-coding for the clustering is shown in the bottom right panel. This figure provides a visual distinction among different transcriptional dynamics, according to the gene’s relative TES abundance throughout the infection. (JPG) [file pone.0320439.s009.jpg]

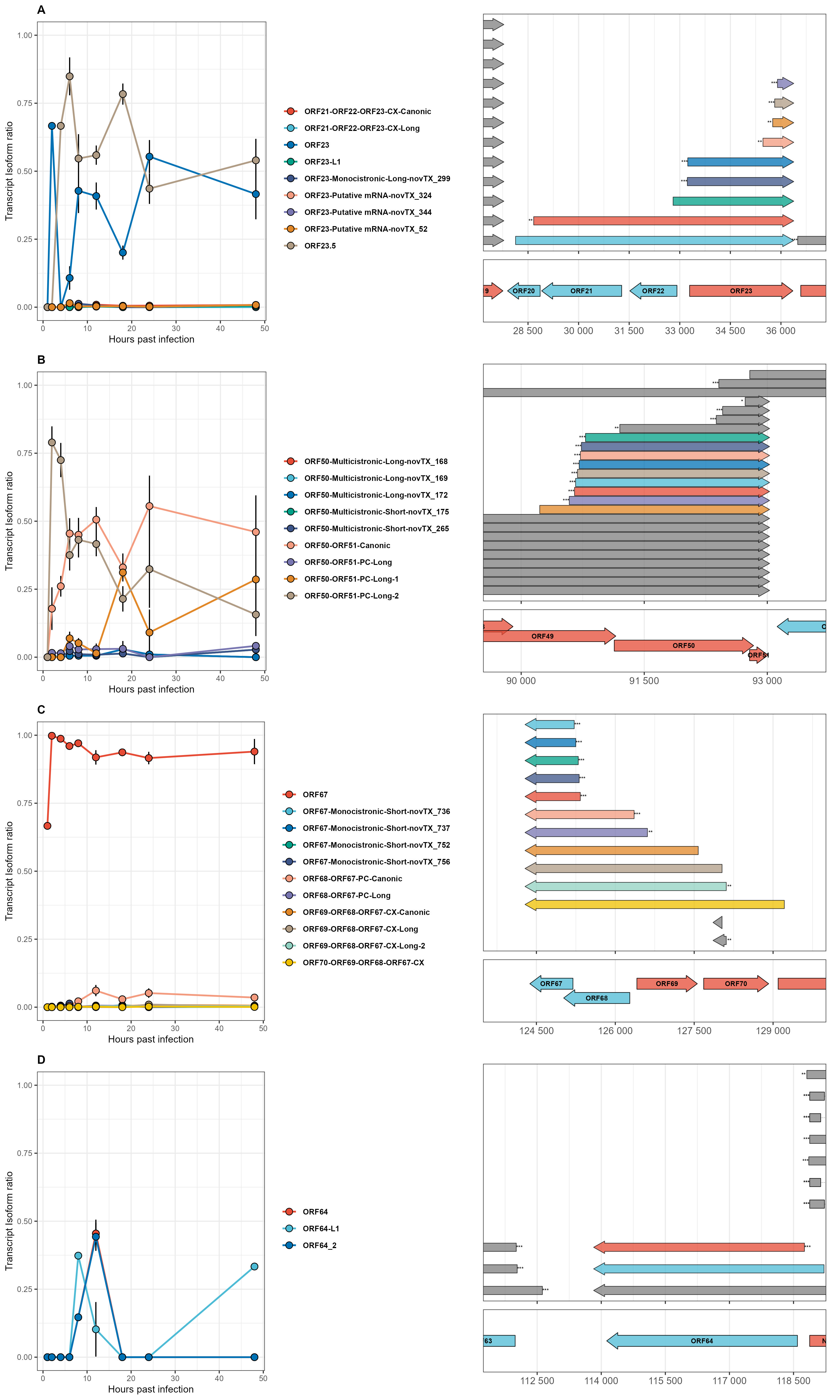

Supplement: S10 Figure — This figure illustrates the dynamics of different transcript isoforms for the selected EHV-1 genes (A) ORF23, (B) ORF51, (C) ORF67, (D) ORF64. The right side of each panel shows the transcript annotations, along with their parent genes and genomic locations displayed below them, with light red indicating positive-strand genes and light blue indicating negative-strand genes. The analysis focused on transcripts that matched exactly, allowing a deviation of +/- 2 nucleotides (nt) for splice junctions and +/- 10 nucleotides for the start and end positions of transcripts. Asterisks indicate the CAGE-Seq significance level for each reference transcript. On the left side of each plot, the temporal trends of these transcript isoforms are depicted, with averages and standard deviations (SD) calculated for each time point post-infection, based on read count data from the dcDNA-Seq. Each data point is linked by lines to demonstrate the progression over time. The transcript isoforms, are color-coded according to their distinct isoforms, with these color matching those used for the points and lines in the left panel. The isoform counts were normalized against the total number of isoform counts for each gene in each sample to calculate the ratio of each isoform. Isoforms on the right side are colored grey, if they not originate from the given gene and thus were not included in the isoform ratio calculation. (JPG) [file pone.0320439.s010.jpg]

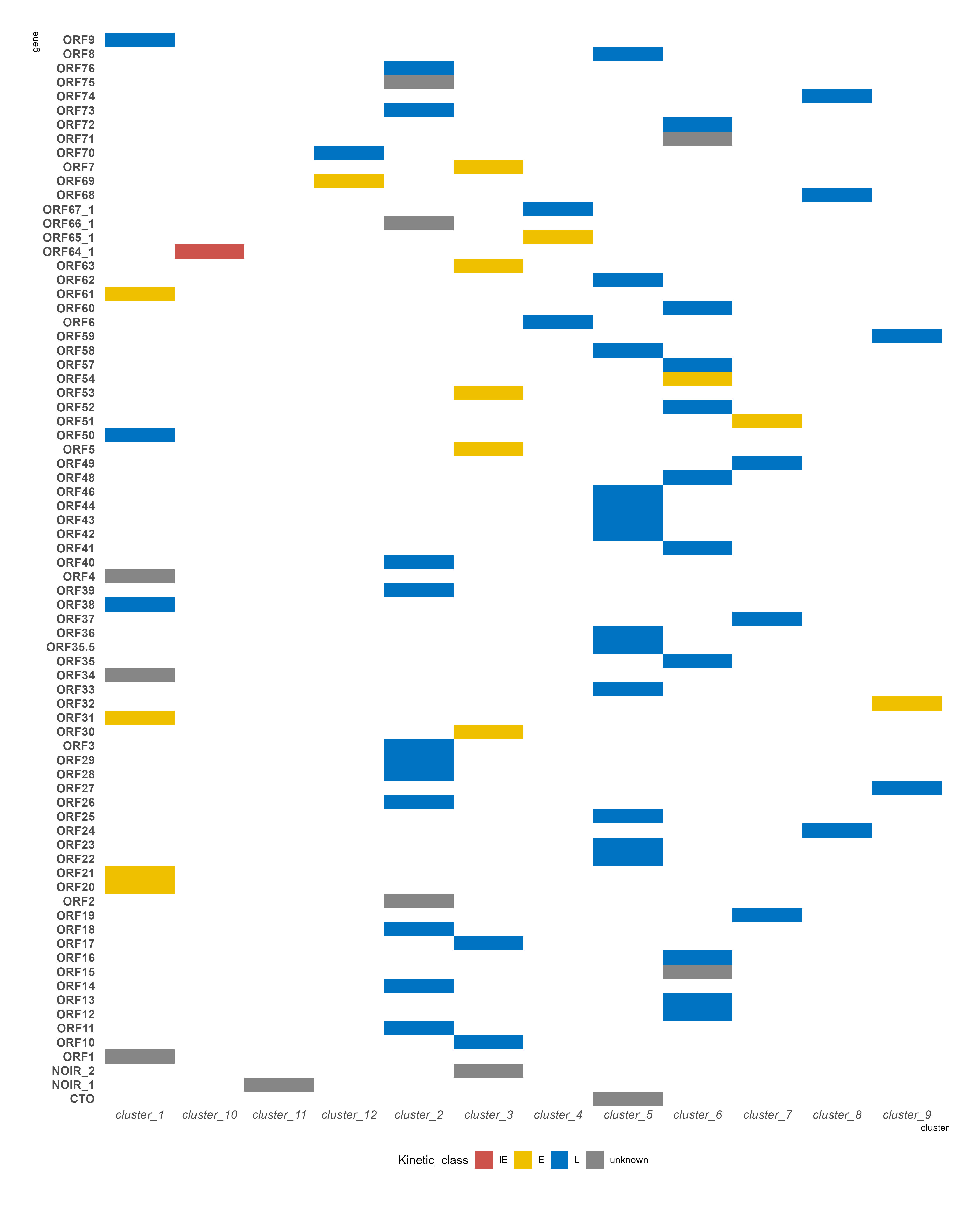

Supplement: S11 Figure — The clustering of EHV genes is based on the normalized canonical transcript counts (reads spanning from the canonical TSS tot the TES), compared to the traditional kinetic classification. The rows show the de novo cluster memberships, while the color represent the kinetic classes. (JPG) [file pone.0320439.s011.jpg]

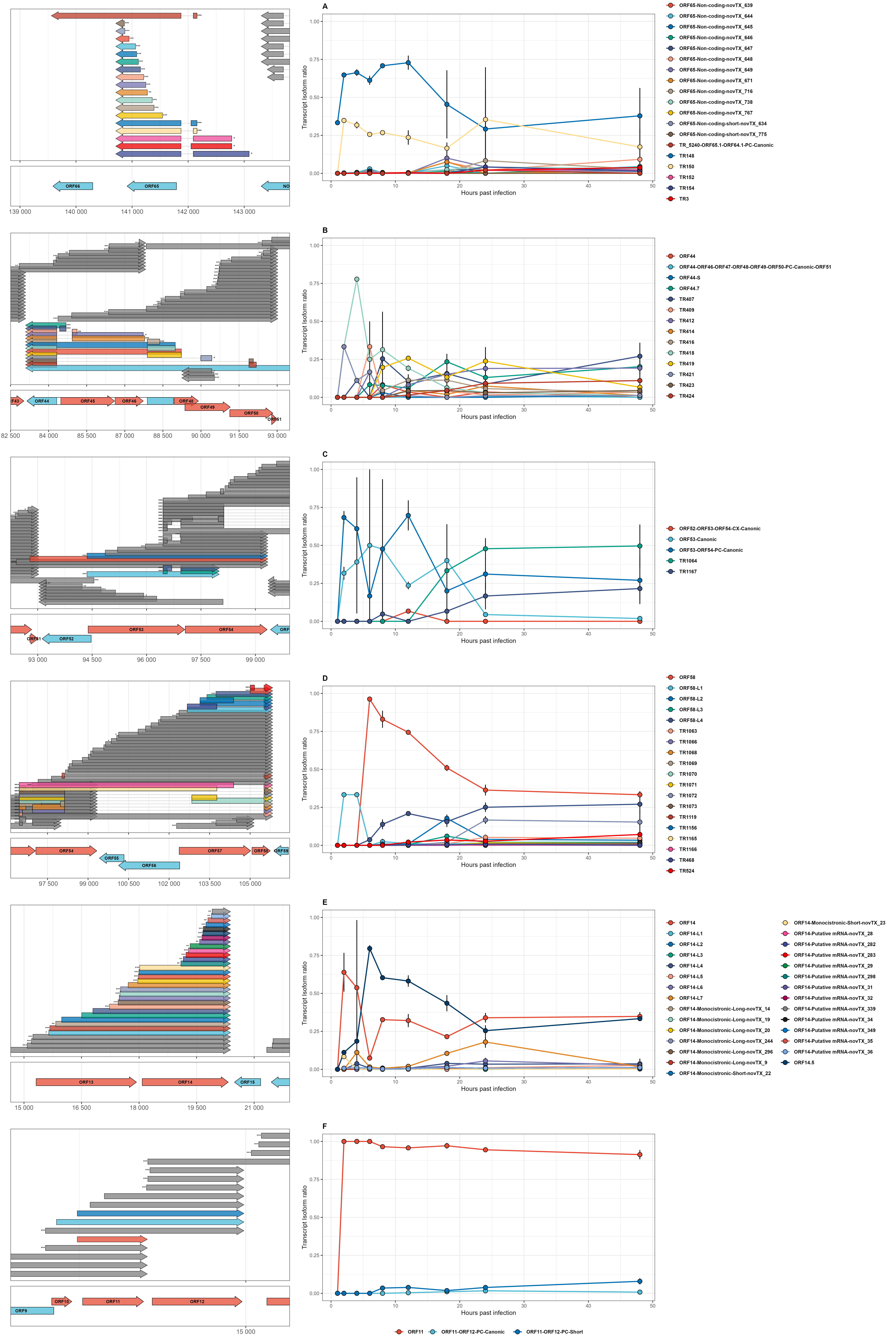

Supplement: S12 Figure — The right side of each panel shows the transcript annotations, along with their parent genes and genomic locations displayed below them, with light red indicating positive-strand genes and light blue indicating negative-strand genes. The analysis focused on transcripts that matched exactly, allowing a deviation of +/- 2 nucleotides (nt) for splice junctions and +/- 10 nucleotides for the start and end positions of transcripts. Asterisks indicate the CAGE-Seq significance level for each reference transcript. On the left side of each plot, the temporal trends of these transcript isoforms are depicted, with averages and standard deviations (SD) calculated for each time point post-infection, based on read count data from the dcDNA-Seq. Each data point is linked by lines to demonstrate the progression over time. The transcript isoforms, are color-coded according to their distinct isoforms, with these colors matching those used for the points and lines in the left panel. The isoform counts were normalized against the total number of isoform counts for each gene in each sample to calculate the ratio of each isoform. Isoforms on the right side are colored grey, if they don’toriginate from the given gene and thus were not included in the isoform ratio calculation. (JPG) [file pone.0320439.s012.jpg]
