# Supplementary material for: Mapping the temporal transcriptomic signature of a viral pathogen through CAGE and nanopore sequencing
Source: PLoS One. 2025 Apr 15;20(4):e0320439. doi: 10.1371/journal.pone.0320439 (PMC11999163; doi:10.1371/journal.pone.0320439)
Supplement: S13 Figure — The annotation of transcripts is based on strict criteria, resulting in the exclusion of a significant number of viral reads. To circumvent this loss, we used the raw sequencing reads to illustrate the true extent of transcriptional overlaps created by genes arranged in divergent and convergent orientations. (PDF) [file pone.0320439.s013.pdf]

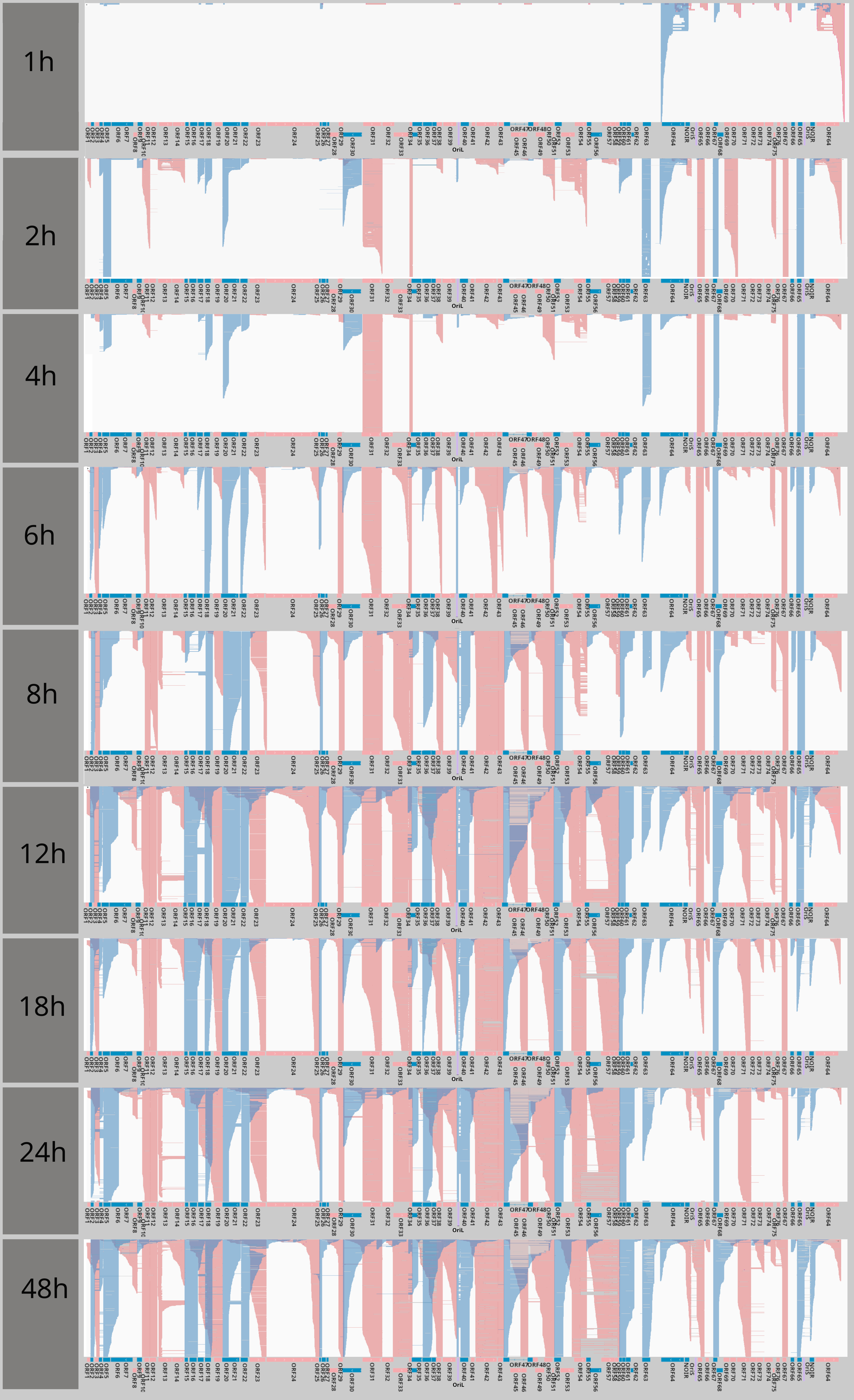
1h
2h
4h
6h
8h
12h
18h
24h
48h
