# Supplementary material for: Mapping the temporal transcriptomic signature of a viral pathogen through CAGE and nanopore sequencing
Source: PLoS One. 2025 Apr 15;20(4):e0320439. doi: 10.1371/journal.pone.0320439 (PMC11999163; doi:10.1371/journal.pone.0320439)
Supplement: S2 Table — (DOCX) [file pone.0320439.s015.docx]

**Concentrations of total RNAs**

| **Time point** | **1h** | **2h** | **4h** | **6h** | **8h** | **12h** | **18h** | **24h** | **48h** |
| --- | --- | --- | --- | --- | --- | --- | --- | --- | --- |
| **ng/µl** | 814 | 702 | 906 | 914 | 788 | 592 | 658 | 308 | 272 |

**Concentrations of poly(A)-selected RNAs**

| **Time point** | **1h** |  | **2h** | **4h** | **6h** | **8h** | **12h** | **18h** | **24h** | **48h** |
| --- | --- | --- | --- | --- | --- | --- | --- | --- | --- | --- |
| **1st elution (ng/µl)** | 8,06 |  | N/A | N/A | 9,94 | 9,38 | 4,6 | 9,24 | N/A | N/A |
| **2nd elution (ng/µl)** | 27,4 |  | 14,2 | 18,3 | 18,5 | 5 | 8,92 | 13,9 | 7,32 | 6,86 |
