# Supplementary material for: Mapping the temporal transcriptomic signature of a viral pathogen through CAGE and nanopore sequencing
Source: PLoS One. 2025 Apr 15;20(4):e0320439. doi: 10.1371/journal.pone.0320439 (PMC11999163; doi:10.1371/journal.pone.0320439)
Supplement: Attachment — Submitted filename: Answers to the reviewers.docx [file pone.0320439.s020.docx]

**Reviewer #1:**

***1)*** *While it is clear why the authors perform CAGE, it remains unclear what the advantage is of now performing dcRNA-seq rather than direct RNA sequencing, which the authors already previously performed. Furthermore, they already published an article performing a re-annotation of the EHV-1 transcriptome (Tombácz, Dóra et al. Heliyon, Volume 9, Issue 7, e17716) but no comparison to this re-annotation is performed. It is thus unclear how their new annotation (the second within a year) compares to the previous one. For instance, they find that “Specifically, 251 transcripts received the highest level of support (***), indicating robust validation by CAGE-Seq. Medium support (**) was found for 47 transcripts, while the lowest level of support (*) was seen in 51 transcripts (Supplementary Table 2).” But it is unclear whether these are novel transcripts detected or previously annotated ones, in particular ones from their previous study.*

We thank the reviewer for the detailed comments and the opportunity to clarify the methodological choices and novel contributions of our study.

Direct RNA sequencing (dRNA-Seq) is valuable for identifying splice sites and transcription end sites (TESs) due to the use of oligodT primers and the low variance in TESs of alphaherpesviruses. However, this technique lacks accurate 5' end information due to 5' truncation caused by motor protein stalling, which limits its reliability in identifying transcription start sites (TSSs). Additionally, dRNA-Seq is prone to various types of errors compared to dcDNA-RNA-Seq (e.g., base-calling issues and read truncation), motivating us to apply a combined approach. Integrating both techniques allows us to achieve more reliable results by leveraging the strengths of each method.

In this study, we employed CAGE-Seq, which is widely accepted for its high resolution in TSS identification, specifically to accurately determine TSSs, complementing the TES and splice site information obtained from dRNA-Seq. While our previous publication focused on annotating canonical transcripts, the current study allowed us to identify additional transcript isoforms and novel TSSs. Here, dcDNA-Seq proved particularly suitable, as it avoids amplification steps and generates full-length reads with true TSSs, thanks to the presence of adapters at the ends of reads (no adapters are used for dRNA-Seq). Thus, dcDNA-Seq provides a more accurate depiction of transcriptional timing.

We appreciate the reviewer’s request for a clearer comparison with our previous annotation. In the revised manuscript, we explicitly distinguish between novel transcripts and those previously detected. While refining the transcriptome annotation is a significant advancement, the primary novelty of our study lies in the comprehensive investigation of temporal transcriptional dynamics, providing new insights into the regulatory complexity of EHV-1. These differences are highlighted in Supplementary Table S2 and the revised text.

***2)*** *Often the conclusions presented in the article are only substantiated with some references to figures or supplementary tables without any explanation on how they come to this conclusion. This applies e.g. to “Subsequently, we performed long-read dcDNA sequencing. Our findings reinforced that orf64 is the sole IE gene in EHV-1 (Supplementary Table 1).” While in this table ORF64 is the only one with high numbers of reads (which are not normalized to transcript length by-the-way), they find reads for other ORFs at lower levels. So, they need to be clearer regarding why they do not consider the other ORFs IE. Notably, it is never explained which time-points would be considered immediate-early, early or late, making it even more difficult to follow the authors’ logic. Another example is the kinetic profiling of EHV-1 Transcripts, which is poorly described. In particular, they perform two different types of normalization (one to viral and one to host reads), without analyzing whether these provide different results or why. It also remains unclear whether the grouping of ORFs is based on the IE, E and L annotation or the new clustering they performed and whether both are consistent. They also do not explain what viral read count-normalized TSS-TES dynamics are for which they perform the clustering.*

In the revised manuscript, we have added explanations to better substantiate our conclusions.

Regarding ORF64, the CHX treatment identified this gene as the sole immediate-early (IE) gene. We concluded this based on two observations from the data in Supplementary Table S1: (1) only ORF64 transcripts are expressed at high levels, and (2) increasing CHX concentration further amplifies the difference in expression between ORF64 and other genes. The remaining genes are expressed at such low levels that they likely represent transcriptional noise, which occurs because CHX does not fully inhibit protein synthesis. This is a normal phenomenon.

We are uncertain about the reviewer’s suggestion to normalize by transcript length, as we are not aware of a statistically validated method tailored for dcDNA-Seq data. Since dcDNA-Seq is performed without amplification or deliberate fragmentation of native RNA, traditional length-based normalization approaches (e.g., RPKM) are not strictly necessary. Although longer transcripts (e.g., ORF64) may be underrepresented as full-length molecules, we accounted for this by analyzing TSSs, TESs, and transcripts independently. In the revised manuscript, we have also clarified the time points associated with IE, E, and L transcripts as requested by the reviewer.

In the revised manuscript, we have included a more detailed explanation of our de novo clustering methodology for TSSs, TESs, and transcripts as well the evaluation of these results. We introduce a new section, *Gene-Level Clustering of Canonical Transcripts,* wherein we discuss in detail these results. After careful consideration prompted by the reviewer’s feedback, we decided to use only viral read counts for normalization. Initially, we explored host-read normalization, but this approach skewed the results because many viral genes increase dramatically over time relative to host transcripts, making direct comparisons less transparent. Using viral reads alone allowed us to capture the relative changes in viral gene expression more accurately, as it provided a consistent reference point within the rapidly changing viral transcript population. We also considered traditional normalization methods, such as those implemented in DESeq2. However, many genes exhibited extremely low counts at certain time points (mainly in 1 and 2 hpi samples), leading to large numbers of zeros. DESeq2 and similar algorithms assume that most genes do not change significantly, an assumption that does not hold in our dataset, where substantial portions of the viral genome undergo marked temporal shifts in expression. As a result, applying these methods would not yield reliable normalization factors. Given these constraints, we chose to normalize to total viral read counts and then cluster genes based on their time-resolved expression patterns. This approach allowed us to identify de novo kinetic classes that better reflect the authentic temporal dynamics of EHV-1 transcription. We now explicitly detail these considerations in the revised text, ensuring that readers understand the rationale behind our normalization strategy and the resulting clustering analyses.

***3)*** *Obvious and important analyses are missing, this includes e.g.*

***3a.*** *A detailed analysis of the link between TSS and TES sites. They find that there is sometimes a discrepancy between the kinetics of TSS sites and TES sites, and they attribute this to the presence of multicistronic ORFs and alternative TES sites for a gene. However, since they performed long read sequencing they can match TSS to TES sites and explicitly analyze whether this is the explanation or is something else is going on.*

We appreciate the reviewer’s suggestion to analyze the link between TSS and TES sites more explicitly. In this study, we identified transcripts containing TSS, TES, and intron information, enabling detailed mapping of these elements. However, for certain TSS sites, precisely validated transcripts remained elusive, likely due to their low abundance or their presence in very long polygenic transcripts, which are often underrepresented in long-read sequencing datasets. In the revised manuscript, specifically in the new section “*Linking TSS and TES Sites*,” we provide additional explanations and references to our analyses that match TSSs to TESs. We also discuss how alternative isoforms and multicistronic arrangements can explain observed discrepancies between TSS and TES kinetics, thereby offering a clearer understanding of these complex transcriptional patterns.

***3b.*** *The dynamics of spliced transcripts expression focuses only on the presence of spliced vs. unspliced transcripts but does not take into account the presence of different spliced variants for the same gene. This needs to be analyzed, whether there is just a general splicing trend during infection or whether particular spliced transcripts.*

We have revised the manuscript to address the expression patterns of different spliced variants within the same gene, rather than only considering spliced vs. non-spliced aggregates. This analysis now shows whether particular spliced transcripts dominate at specific time points, offering a more nuanced view of splicing dynamics during infection.

***Some other issues:***

***1)*** *The manuscript is written in a very confusing manner. In particular, the outline of the study is only described at the beginning of the results rather than at the end of the introduction, which makes the methods section difficult to understand. There is also no explanation on why they do not use direct RNA sequencing as in their previous study but need dcDNA-seq here.*

We have thoroughly revised the manuscript to improve clarity and have restructured the introduction to better outline the study’s objectives and methodology.

Regarding the choice of dcDNA-seq over direct RNA sequencing (dRNA-Seq) in this study, we opted for dcDNA-seq as it provides certain advantages in accurately identifying transcription start sites (TSSs) and full-length transcript structures. Unlike dRNA-Seq, which often truncates the 5' end due to motor protein stalling, dcDNA-seq avoids this issue by producing full-length reads with high reliability. This method also eliminates the need for amplification, preserving the original transcript proportions, which is crucial for accurately assessing transcriptional dynamics. Therefore, dcDNA-seq allowed us to achieve more robust and comprehensive results for the goals of this study. In addition, since dRNA-Seq cannot be barcoded, but dcDNA can be, we were able to sequence a total of 27 samples ranging from hpi1 to 48, a total of 9 time points in triplicates.

***2)*** *Upper- and lower case is used inconsistently for ORFs and genes.*

We have revised the manuscript and now consistently use capital letters (ORF).

***3)*** *The noir/NOIR gene/transcript appears to be a novel transcript from their previous study, but that is never explained.*

We have revised the manuscript to clarify the NOIR transcripts’ origin and their relationship to our previous findings in the *Comparison of replication origin-associated transcripts of EHV-1 and PRV* section.

***4)*** *The font size in figures is generally very small, making it often almost impossible to discern the ORFs they refer to in the manuscript and at least partially confirm their conclusions. Furthermore, x- and y-axis labels are often missing as well as color legends within the figures. While sometimes this is explained in the caption to the figures, it is not done so consistently and makes it difficult to understand the figures.*

We have improved figure quality, increasing font sizes and adding clearer axis labels, legends, and color indicators. This makes it easier to confirm our conclusions visually.

***5)*** *Their “in-house developed R pipeline” should be made available either as supplement or Zenodo/Github.*

We have made it our complete analysis and code to generate the plots available on github.

***6)*** *It is unclear why they explicitly note some R packages but not the “other R-packages from the Bioconductor repository”.*

We have clarified our mention of software tools and R packages, providing a consistent explanation of all packages used, including those from Bioconductor.

***7)*** *I do not understand the point of Figure 6.*

This figure illustrates raw reads, revealing a much more complex transcriptome structure compared to one based solely on annotated transcripts. The main takeaway of the figure is that RNA molecules exhibit extensive transcriptional overlap; which likely impacts gene expression regulation as transcriptional machineries may interfere within these overlapping regions. We have elaborated on this topic in the revised manuscript.

**Reviewer #2:**

*This is a fine sequencing paper for equine herpesvirus. It is well written and is in line with the large volume of stellar sequencing manuscripts published by this group. I appreciate their work. However, it would be nice to use proteomic to define the alternatively spliced variants of the transcriptome of herpesviruses. So a transcriptome and proteome manuscript would be even better.*

We thank the reviewer for the valuable comments and constructive feedback on the manuscript. The suggestion to incorporate a proteomic analysis is well-noted, and we fully recognize the importance of such an approach. However, including this analysis would significantly broaden the scope of the current study. Therefore, while this manuscript does not intend to address proteomics, we plan to consider this in a dedicated future study, where the complexity of proteomic data can be thoroughly explored. Thank you once again for the helpful insights, which have contributed to refining the research direction.
