# Supplementary material for: Mapping the temporal transcriptomic signature of a viral pathogen through CAGE and nanopore sequencing
Source: PLoS One. 2025 Apr 15;20(4):e0320439. doi: 10.1371/journal.pone.0320439 (PMC11999163; doi:10.1371/journal.pone.0320439)
Supplement: Attachment — Submitted filename: PlosOne-answer-to-the-Reviewer.docx [file pone.0320439.s021.docx]

**Reviewer #1**: *The authors addressed most of my previous concerns, however there are some issues, which require more detail/precision in the manuscript.*

*In the order of appearance in the text:*

*- "we used an in-house developed R pipeline." -> Here, they should include the link to the github repository. The link in the data availability section is not working though.*

The link has been corrected and should work now.

*- "The CAGEfightR [35] package was used to determine TSS positions. The TSS clusters within a 10 nucleotides window were termed identical." Does TSS cluster refer to the TSS positions outputted by CAGEfightR or are these the clusters obtained after merging TSS within 10 nt.*

TSS clusters are groups of transcription start sites (TSSs) that are identified and merged within a 10-nucleotide window by CAGEfightR, rather than individual raw TSS positions. The software first detects individual CAGE TSSs (CTSSs) and then groups them into clusters based on the specified distance threshold.

*- "Reference transcript counting": It is unclear what is compared against the reference annotation here. The reads from dcRNA-seq? This needs to be clarified.*

We introduced the term *transfrags* to facilitate understanding of the workflow. We use transfrags to refer to a list of unique alignments of the dcDNA reads to the viral genome, considering both exons (matches in the alignment) and introns (appearing as Ns at the nucleotide level). Their characteristics and distribution throughout the dcDNA dataset were used as the basis for downstream analysis. We have clarified that reference transcript counting was performed by comparing the list of assembled transfrags (from dcDNA-Seq) to our previously annotated transcript dataset using GFF-compare. Thus, the comparison was not performed using individual sequencing reads but rather by aligning the transfrags to our reference transcript list to quantify the presence of reference transcripts in each sample.

*- nucleotide vs. nt-s: The authors use both terms, but should only use one consistently*

We have standardized the terminology and now consistently use the term 'nucleotide' throughout the revised manuscript

*- "The clusters were merged with the dcDNA-Seq dataset" -> The TSS clusters?*

Yes, this refers to the TSS clusters obtained from CAGEfightR. For clarity, we have explicitly stated 'TSS clusters' in the revised text.

*- ". , which contains transcript identities based on the alignment of 5′-ends" -> I don't understand what this is supposed to mean or refer to. The TSSs or the dcRNA-seq, if the latter why would only the 5'end be aligned. This needs to be rephrased to be unclear*

This refers to the TSS clusters obtained from CAGEfightR and the transfrags (derived from the dcDNA data). It means that the two datasets were merged based on the genomic position (start, end, and strand) of the former and the 5′-end position (and strand) of the latter. This has been made clearer in the revised article by explicitly using the term 'transfrags'.

*- "Transcripts were reconstructed by pairing validated TSS peaks": what are validated TSS peaks? peaks confirmed by dcRNA-seq in the previous paragraph? But this already merged transcripts (from dcRNA-seq? unclear) with TSS, why do you need to do it again? Also transcripts are from the dcRNA-seq data or where do they come from?*

CAGE TSS clusters were sometimes broad, exceeding 150 nucleotides in certain cases. To enhance precision, we refined these TSSs using the 5′-end counts of transfrags derived from dcDNA-Seq. For each TSS cluster, peak analysis was performed to identify validated TSS peaks, which differed from the original CAGE TSS clusters but were supported by both CAGE-Seq and dcDNA-Seq data. This refinement ensured that only high-confidence TSSs were used for novel transcript reconstruction. While some transfrags could not be assigned to reference transcripts from our previous study, their strong TSS signals in both dcDNA-Seq and CAGE-Seq indicated that they likely represented previously unannotated transcripts. The full workflow has been clarified in the Methods section, and the correct terminology is dcDNA-Seq, not dcRNA-Seq.

*- "their 3′-ends overlapped a known TES" -> known means from the previous annotation?*

Yes, 'known' refers to TESs from our previous transcriptome annotation. This has been explicitly clarified in the revised text.

*- "This approach enabled the integration of the CAGE-Seq and dcDNA-Seq datasets to annotate TSSs."-> but you did this already in the previous section? Why did you do this again?*

This referred to the previous section. We have clarified this distinction in the text.

*- "Newly assembled transcripts were integrated with our prior annotation"-> how?*

Using the gff-compare output, we analyzed the relationship between each novel transcript and the reference list. Novel transcripts that met the validation criteria - TSS-TES pairing, expression threshold, and support from multiple sequencing methods - were incorporated into the updated annotation. Additionally, we manually reviewed the newly identified transcripts and compared them to our previous transcript list.

*- "Validated transcripts"-> when is a transcript considered validated?*

A novel transcript (not found in the reference list within the ±10 nucleotide wobble) was considered validated if it met all of the following criteria:

- Supported by at least three independent dcDNA-Seq reads.
- The TSS was within ±10 nt of a validated TSS peak (within a CAGE-Seq TSS cluster).
- The TES overlapped a known TES within ±10 nt.
- Correct 5′- and 3′-adapter sequences were detected.

This definition has been explicitly included in the Methods section.

*- "To further filter TSS transcripts" -> what are TSS transcripts, it has not been defined to which transcripts this term refers to.*

This was a typographical error; we were referring to 'To further filter the TSS of transcripts …'. This indicates that we applied stricter criteria when accepting 5′-truncated transcripts containing 5′-truncated ORFs to eliminate sequencing artifacts. These artifacts occurred when the reverse transcription step in the dcDNA protocol was incomplete. The error has been corrected.

*- "To identify groups of TSSs, TESs, and transcripts with similar temporal expression patterns, we performed de novo clustering on normalized gene expression data. [..] For gene clustering, .." -> gene clustering aims to identify groups of transcripts with similar temporal expression patterns? If not how are these groups of transcripts identified. This needs to be clarified.*

Yes, transcripts were grouped into clusters based on their temporal expression profiles. The terms *gene expression* and *transcript expression* were used interchangeably, but we have clarified that this analysis specifically refers to transcript expression, as only canonical transcripts were counted and clustered. Unlike gene-level clustering, which includes all transcript isoforms, this approach eliminates ambiguity in assigning isoforms to specific genes. Differential transcript expression, including isoform-specific patterns, was analyzed separately in the sections *'Dynamics of transcriptional isoform switching in selected genes*' and *'Dynamics of spliced transcript expression*'. These distinctions have now been explicitly clarified in the Methods section.

*- "Our findings reinforced that ORF64 is the sole IE gene in EHV-1" -> It needs to be explicitly mentions that ORF64 is the only gene with significant expression levels after CHX treatment.*

We have revised the manuscript to explicitly state that ORF64 is the only gene with significant expression levels following CHX treatment. Statistical analyses—including t-tests, Z-score analysis, coefficient of variation (CV), and interquartile range (IQR) outlier detection—confirm that ORF64 is the sole viral gene significantly expressed under CHX treatment, while other detected transcripts likely represent background noise. This finding reinforces ORF64’s classification as the only true immediate-early (IE) gene in EHV-1.

*- "Among the examined transcripts, 251 received the highest level of support (***) - indicating robust validation - while 47 had medium support (**) and 51 showed the lowest level of support (*) (see Methods for details)." -> Methods only talks about assigning confidence to TSS. Are you talking about TSS here? You need to be more precise with your terms!*

Here, we refer to previously published transcripts - specifically, how many received certain confidence categories in our CAGE-based TSS analysis. As described in the Methods (TSS Clusters Validation), the ‘’, ‘**’, and ‘’ levels apply exclusively to TSS clusters identified via CAGE-Seq. We then merged these clusters with the dcDNA-Seq transfrags, to which reference transcripts had already been assigned by gff-compare based on their 5′-position overlaps. This clarification has been added to the *Transcript Merging and TSS Refinement* section. The reported numbers - 251 (***), 47 (**), and 51 (*) - represent reference (previously annotated) transcripts that inherited TSS confidence ratings from CAGE-Seq. However, if a transfrag did not align with any known reference transcript but exhibited a strong CAGE signal and a correct dcDNA 5′-adapter signal, we assembled a novel transcript from that transfrag. This process is now detailed in the *Transcript Assembly and Validation* section.

*- OriS = Oris? If yes, please use a consistent notation, if not explain.*

OriL and OriS are the correct terms, and we have updated the article accordingly.

*- Supplementary Figure S1 needs to indicate the location of raRNA, Ori-L and the other genes/mRNAs mentioned in this section, otherwise it cannot be understood.*

In Supplementary Figure S1, the aligned nucleotide sequences of the NOIR (A) and CTO (B) genes in the three viral strains are shown along with their orientation. Since this represents a multiple sequence alignment of genes, including surrounding genomic regions and/or transcripts was not feasible. To improve clarity, we have created two additional figures. Supplementary Figure S2 provides a high-resolution view of the EHV-1 replication origin region, while Supplementary Figure S3 presents the same for PRV. These figures clearly depict the locations of the NOIR and CTO genes, their corresponding transcripts, and the replication origins within the given region. In these figures, molecules overlapping the replication origin are shown in green, those transcribed from the forward strand are in red, and those transcribed from the reverse strand are also in green.

*- noir = NOIR? If yes, please use a consistent notation, if not explain.*

Any instance of 'noir' has been omitted; we now use 'NOIR' consistently throughout the manuscript.

*- Legend to Supplementary Figure S2 and Supplementary Figure S5 need to state the scale of the y-axis is determined independently for each time-point*

We have revised the legends for both Supplementary Figure S2 (now S4) and Supplementary Figure S5 (now S6) to explicitly state that each facet’s y-axis is scaled independently (using ggplot’s scale = "free_y"). This ensures that the y-axis range adapts to the data in each time-point facet, allowing for clearer visualization of expression profiles.

*- Supplementary Figure S4 should be before Supplementary Figure S3 und referenced in the previous paragraph, which discusses the traditional kinetic clusters*

The reviewer is correct once again; we have reordered Supplementary Figures S3 (now S5) and S4 (now S6). Additionally, following the same logic, we also reordered Supplementary Figures S6 (now S8) and S7 (now S9).

*- Figure 7 should be a Supplementary Figure as it is too large for the main manuscript. The text will be unreadable if it is scaled to fit in the main manuscript.*

We agree. Figure S7 has been renumbered as Supplementary Figure S13 to improve the visibility of annotations and other text.

*- "By integrating multiple data sources (dcDNA-Seq, dRNA-Seq, and CAGE-Seq) and using different tools - including LoRTIA for dcDNA-Seq libraries and NAGATA for dRNA-Seq data" -> where is the dRNA-seq data integrated? This did not become clear in the methods (see also my above questions).*

We utilized our previous dRNA-Seq dataset and the NAGATA software to (1) validate introns and (2) confirm the presence of newly identified TSSs, with a specific focus on 5′-truncated ORF-carrying 'putative transcripts.' This approach was primarily aimed at minimizing the risk of annotating sequencing artifacts caused by enzyme stalling during reverse transcription, a known issue in dcDNA-Seq. These validation steps were undertaken to complement and verify both the sequencing methods used in this study (dcDNA-Seq and CAGE-Seq) and the bioinformatic approach.

In the Methods section (see Filtering 5′-Truncated ORF-Carrying Transcripts and Transcript Assembly and Validation), we detail how we reanalyzed our earlier dRNA-Seq data with NAGATA software to ensure that 5′-truncated ORF isoforms—and the introns identified in dcDNA-Seq—were also supported by dRNA reads. Specifically:

Intron Validation: Introns detected in dcDNA-Seq were accepted only if they also appeared in our previous dRNA-Seq dataset.

TSS Cross-Checking: Any novel TSS identified in dcDNA-Seq and CAGE-Seq had to be detectable within a 25-nt window in the dRNA-Seq-NAGATA output to confirm its authenticity.

This integrated approach helped eliminate potential artifacts and reinforced our confidence in newly discovered TSSs and alternative isoforms."

*- "The R codes used to perform the analysis and generate the plots are available at: https://github.com/Balays/EHV-1-dynamic" -> the link does not exist.*

The provided link *<https://github.com/Balays/EHV-1-dynamic>* is now functional, and the scripts used to generate the analysis and figures are accessible.
